# Brain Abscess and Keratoacanthoma Suggestive of Hyper IgE Syndrome

**DOI:** 10.1155/2015/341898

**Published:** 2015-04-28

**Authors:** Soheyla Alyasin, Reza Amin, Alireza Teymoori, Hamidreza Houshmand, Gholamreza Houshmand, Mohammad Bahadoram

**Affiliations:** ^1^Department of Pediatrics, Division of Immunology and Allergy, Allergic Research Center, Shiraz University of Medical Science, Shiraz 7134845794, Iran; ^2^School of Medicine, Department of Neurosurgery, Ahvaz Jundishapur University of Medical Sciences, Ahvaz 6135715794, Iran; ^3^Department of Pharmacology and Toxicology, Pharmacy School, Ahvaz Jundishapur University of Medical Sciences, Ahvaz 6135715794, Iran; ^4^Medical Student Research Committee and Social Determinant of Health Research Center, Ahvaz Jundishapur University of Medical Sciences, Ahvaz 6135715794, Iran

## Abstract

Hyper immunoglobulin-E (IgE) syndrome is an autosomal immune deficiency disease. It is characterized by an increase in IgE and eosinophil count with both T-cell and B-cell malfunction. Here, we report an 8-year-old boy whose disease started with an unusual skin manifestation. When 6 months old he developed generalized red, nontender nodules and pathologic report of the skin lesion was unremarkable (inflammatory). Then he developed a painless, cold abscess. At the age of 4 years, he developed a seronegative polyarticular arthritis. Another skin biopsy was taken which was in favor of Keratoacanthoma. Laboratory workup for immune deficiency showed high eosinophil count and high level of immunoglobulin-E, due to some diagnostic criteria (NIH sores: 41 in 9-year-olds), he was suggestive of hyper IgE syndrome. At the age of 8, the patient developed an abscess in the left inguinal region. While in hospital, the patient developed generalized tonic colonic convulsion and fever. Brain computed tomography scan revealed an abscess in the right frontal lobe. Subsequently magnetic resonance imaging (MRI) of the brain indicated expansion of the existing abscess to contralateral frontal lobe (left side). After evacuating the abscesses and administrating intravenous antibiotic, the patient's condition improved dramatically and fever stopped.

## 1. Introduction

Hyper immunoglobulin-E syndrome (HIES) is a rare primary immunodeficiency disease, characterized by the classical triad of recurrent staphylococcal skin abscesses, pneumonia with pneumatocele formation, and elevated levels of serum IgE, usually over 2,000 IU/mL [1]. HIES is a group of primary immunodeficiencies with overlapping and distinct features most frequently caused by deficiency in STAT3 or DOCK8. New hyper IgE syndrome entities have also been reported [1]. These include impairment of PGM3 function (phosphoglucomutase 3) and an enzyme in the glycosylation pathway (glycosylation defect). Such deficiencies are believed to be the genetic cause of hyper IgE syndrome in patients who do not carry mutations in STAT3 or DOCK8 [2].

DOCK8 hyper IgE syndrome patients present in infancy with severe atopic dermatitis and can later go on to develop severe food allergy with positive skin prick test result and specific IgE to food allergens. T helper 2 cell numbers and cytokines were significantly increased in DOCK8 IgE syndrome and atopic dermatitis patients, compared to STAT3 hyper IgE syndrome patients [3].

Particular progress has been made in deciphering the relevance of STAT3 and DOCK8 for B-cell, T-cell, and natural killer cells immunity as well as in understanding allergic features. Multisystemic features of STAT3 deficient hyper IgE syndrome, for example, are recurrent fractures and osteopenia and high degree of vasculopathy and brain with matter hyper intensities. IgG replacement may add to the clinical care in STAT3-deficient hyper IgE syndrome. In DOCK8-deficient hyper IgE syndrome the high mortality and deaths in early age seem to justify allogenic hematopoietic stem cell transplantation [1].

Both dominant and recessive forms have been reported. Autosomal dominant hyper IgE syndrome is almost always caused by dominant negative heterozygous mutations in the gene encoding STAT3. Specific IgE values, skin prick test, and T-cell subset of STAT3 hyper IgE syndrome patients' properties were analogous to those of healthy individuals except for decreased TH17 cell counts [3]. Patients with autosomal dominant hyper IgE syndrome have a history of staphylococcal abscess. Persistent pneumatoceles develop as a result of recurrent pneumonias. Pruritic dermatitis with eczema like skin lesions occurs. Coarse facial features and high incidence rates for scoliosis and hyperextensible joints also are noticeable. Only one patient had a mutation in the gene encoding Tyk2; all of the other reported patients with autosomal recessive hyper IgE syndrome had mutations in the gene encoding DOCK8. DOCK8 may be important for the formation of the immunologic synapse that leads to T-cell activation. A large majority of patients have severe asthma and food allergies. They also could have recurrent skin viral infections, including severe herpes simplex, herpes zoster, and other viral infections. In addition, patients can have abscesses, candidiasis, upper respiratory infections, and pneumonia. Neurologic problems, including strokes, meningitis, and aneurysms, are prominent. Malignancies are also common [1]. Since many genes and cell types are involved in the pathogenesis of this disease, different clinical manifestations have been reported. Reporting of unusual course and presentation of disease helps improve the knowledge of course of the disease. The following case presented with an unusual skin lesion had multiple episodes of skin abscess formation, keratoacanthoma, and brain abscesses.

## 2. Case Report

**Figure 1:**
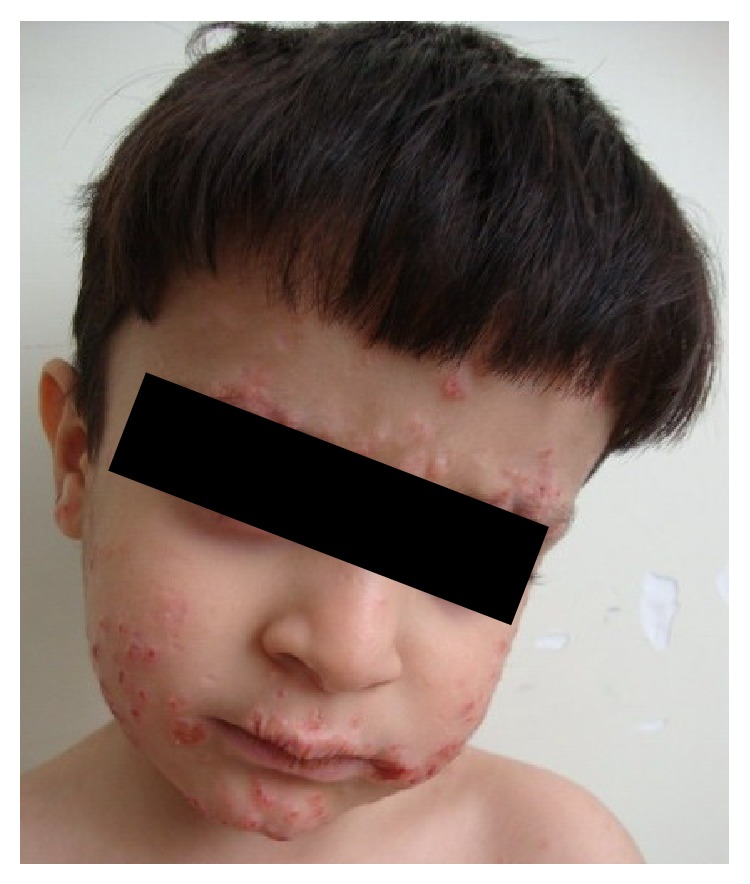
Skin manifestations of keratoacanthoma in hyper IgE syndrome (4 years old).

**Figure 2:**
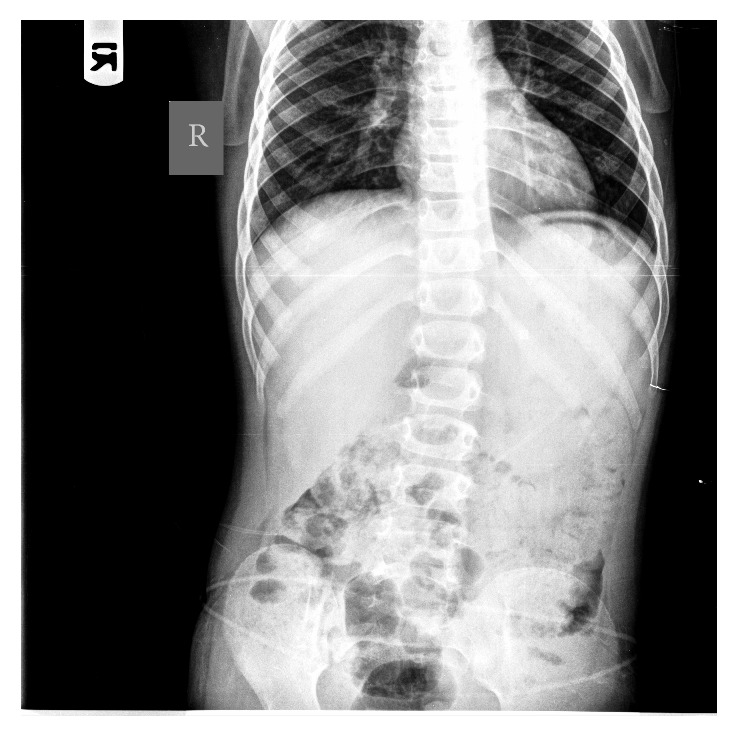
Flat upright X-ray that shows normal chest and scoliosis in thoracolumbar region.

**Figure 3:**
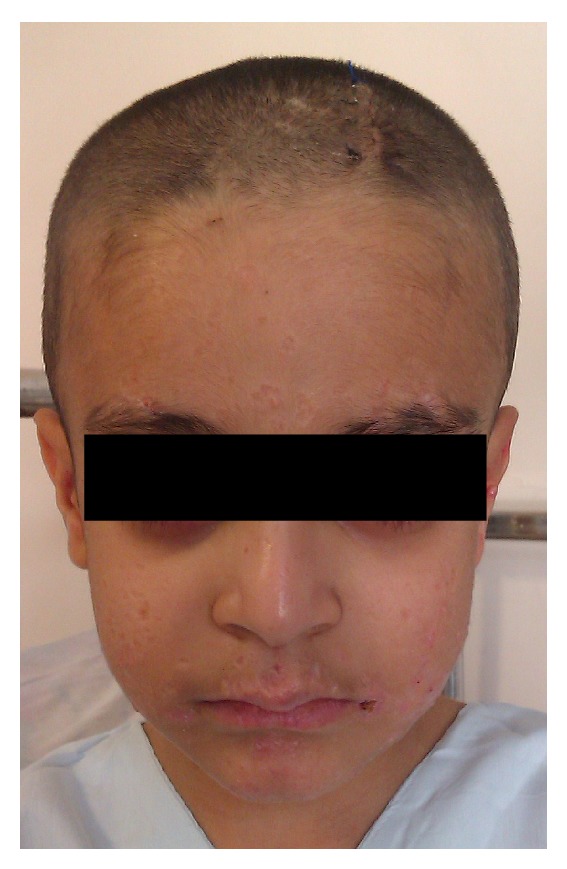
Scars induced after resolution of keratoacanthoma (8 years old).

**Figure 4:**
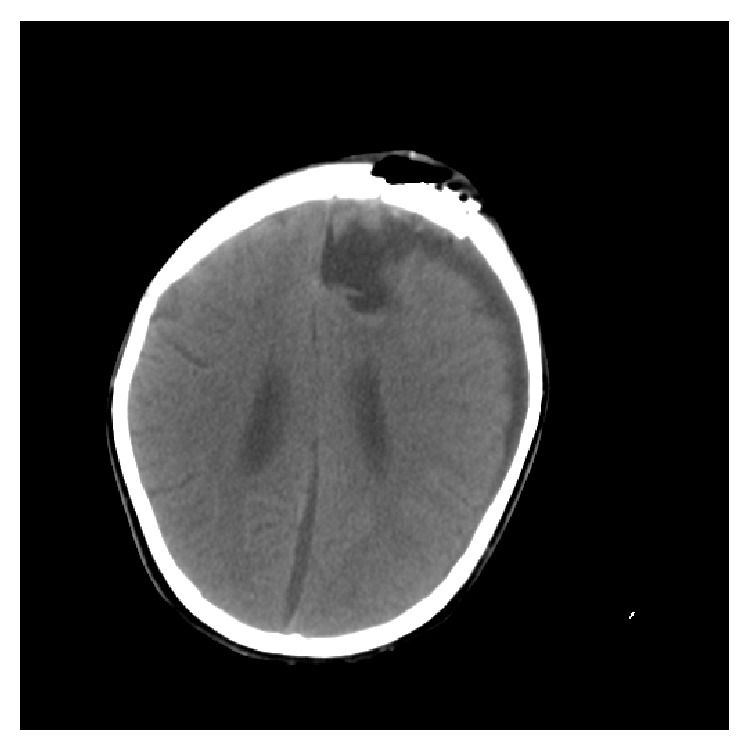
Primary brain CT scan that shows unilateral frontal brain abscess (confined to one hemisphere).

**Figure 5:**
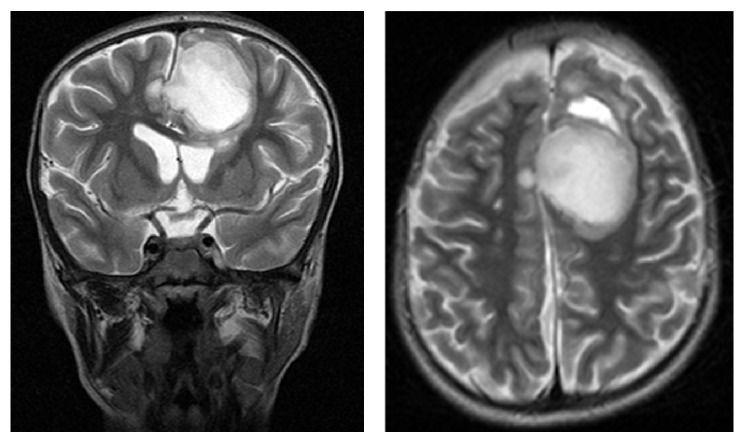
Brain MRI showing expansion of aspirated brain abscess to contralateral frontal lobe.

Our patient is an 8-year-old boy whose disease started with an unusual skin manifestation and extraordinary findings were seen during the course of treatment. At 6 months old he developed generalized red, nontender nodules. At the time, the patient had no systemic manifestation of any disease; therefore only biopsy of the lesion was taken. First biopsy was taken when he was 6 months old; the pathologic report of this biopsy was nonspecific inflammatory process. He developed a painless, cold abscess in the medial axis of his thigh at the age of 2. At that time patient had no abnormal findings in the physical examination or laboratory workup. Thus treatment for a simple abscess was done. At the age of 4, he developed a seronegative polyarticular arthritis which included proximal interphalangeal joints of hands, right elbow, both hip joints, and left knee which responded well to usual treatment for juvenile arthritis. The patient was on daily oral prednisolone and folic acid and weekly oral methotrexate therapy. His ANA level was on normal range. During the same year, another skin biopsy was taken which was in favor of keratoacanthoma (Figure 1), and it also showed wart infection. Multiple eruptive keratoacanthomas of the patient responded well to oral isotretinoin therapy. At this time workup for immune deficiency disease was repeated. A review of family history revealed that the patient's parents were cousins. In addition, workup detected high eosinophil count in complete blood count and high level of immunoglobulin-E but due to financial limitations genetic study was not performed. According to some diagnostic criteria (the National Institute of Health clinical feature scores: 41 in 9-year-olds), he was suggested as hyper IgE syndrome patient (Table 1, Figure 2) [4]. At the age of 8, our patient developed an abscess in the left inguinal region and subsequently he was admitted to the hospital. Complete physical examination was done and nothing except left side inguinal abscess, scars of previous skin lesions, and retained primary teeth was detected (Figure 3). In ultrasonography a collection was detected in subcutaneous region. So, treatment was started by draining the abscess and administering broad spectrum intravenous antibiotics. Few days after admission, the patient developed a nonspecific abdominal pain. Abdominal computed tomography showed mild-free fluid with no abscess formation; also an asymptomatic neural cyst at the root of T10 nerve and outside the spinal canal was seen. The abdominal fluid was not purulent and had no signs of malignancy. During hospitalization, the patient developed generalized tonic colonic convulsion and a fever with no neurologic deficits. Brain computed tomography scan showed an abscess measured 4.6 × 3.3 cm in the right frontal lobe (Figure 4). The abscess was then aspirated. The aspirate showed no evidence of bacterial or fungal infections and pathologic report showed tissue inflammation with inflammatory cells. Gram stain and cultures for bacteria, fungus, and mycobacteria were all negative as well as polymerase chain reaction for mycobacteria and fungus. Patient was febrile for another 2 weeks so we employed broader spectrum antibiotics and IV-IG. After a week passed with no improvement in his condition, a magnetic resonance imaging (MRI) of brain was performed which showed expansion of existing abscess to contralateral frontal lobe (left side) (Figure 5); hence full evacuation of the contents and wall of abscess was done. Repeatedly, diagnostic studies for bacterial, fungal, and mycobacterial infections were negative. After evacuating the abscess, patient's condition improved dramatically and fever stopped. The patient was given intravenous antibiotic for 4 weeks without further complications. In followups, the patient was visited monthly with no neurologic deficits or fever seen.

## 3. Discussion

Hyper IgE syndrome is a rare, primary, complex immunodeficiency disease which results from dysfunction of both T-lymphocytes and B-lymphocytes [1]. This disease was first named as hyper IgE syndrome by Buckley et al. upon observing an association between recurrent staphylococcal abscess formation, chronic eczema, and high level of IgE in blood circulation [5]. Pathognomonic findings in these patients are the presence of pneumatocele. Other respiratory system associated infections include paranasal sinusitis and otitis media [6–8]. Freeman et al. described a case series of 6 hyper IgE patients who died due to fungal and pseudomonas infection of lung [9].

Autoimmune diseases are another symptom of primary immune deficiency diseases and may invade joints and cause arthralgia and arthritis. Joint involvement is more commonly seen in humoral immunodeficiency diseases other than hyper IgE [10]. Brain abscess is an unusual and lethal infection; it usually presents as a space-occupying lesion and is accompanied by headache, nausea, vomiting, lethargy, stupor, and seizure [11]. Immunodeficiency is a risk factor for brain abscess formation. Gatz SA and colleagues reported brain abscess in girls with hyper IgE syndrome as a complication of bone marrow transplantation [12]. Metin et al. described tuberculous brain abscess in patient with hyper IgE syndrome [13]. Also from Iran Amini and colleagues on Tanaffoss 2010 reported brain abscess in patients with hyper IgE syndrome [14]. Among immune deficiency diseases, common variable immunodeficiency is the most common. Though rare, brain abscess is most commonly seen among CVID patients [15]. Abscess formation is one of the manifestations seen commonly in hyper IgE syndrome and occurs mostly in organs such as skin and deep viscera and predominantly in lungs. Nonetheless, brain abscess in association with hyper IgE syndrome has rarely been reported [16].

## Figures and Tables

**Table 1:** Assessment by NIH scoring system with clinical and laboratory tests [4].

Clinical and laboratory finding	Results	Points
Highest serum IgE level (IU/mL)	1,001–2,000	8
Skin abscesses	1-2	2
Pneumonia (episodes over lifetime)	None	0
Parenchymal lung anomalies	Absent	0
Retained primary teeth	>3	8
Scoliosis, maximum curvature	15°–20°	4
Fractures with minor trauma	None	0
Highest eosinophil count (cells/*μ*L)	>800	6
Characteristic face	Mildly present	2
Midline anomaly	Absent	0
Newborn rash	Absent	0
Eczema (worst stage)	Mild	1
Upper respiratory infections per year	1-2	0
Candidiasis	Fingernails	2
Other serious infections	Severe	4
Fatal infection	Absent	4
Hyperextensibility	Absent	0
Lymphoma	Absent	0
Increased nasal width	<1 SD	0
High palate	Absent	0
Young-age correction	>5 years	0

Total		41
